# Excess Mortality During the COVID-19 Pandemic in Jordan: Secondary Data Analysis

**DOI:** 10.2196/32559

**Published:** 2021-10-07

**Authors:** Yousef Khader, Mohannad Al Nsour

**Affiliations:** 1 Department of Public Health Faculty of Medicine Jordan University of Science and Technology Irbid Jordan; 2 Global Health Development Eastern Mediterranean Public Health Network Amman Jordan

**Keywords:** COVID-19, excess mortality, pandemic

## Abstract

**Background:**

All-cause mortality and estimates of excess deaths are commonly used in different countries to estimate the burden of COVID-19 and assess its direct and indirect effects.

**Objective:**

This study aimed to analyze the excess mortality during the COVID-19 pandemic in Jordan in April-December 2020.

**Methods:**

Official data on deaths in Jordan for 2020 and previous years (2016-2019) were obtained from the Department of Civil Status. We contrasted mortality rates in 2020 with those in each year and the pooled period 2016-2020 using a standardized mortality ratio (SMR) measure. Expected deaths for 2020 were estimated by fitting the overdispersed Poisson generalized linear models to the monthly death counts for the period of 2016-2019.

**Results:**

Overall, a 21% increase in standardized mortality (SMR 1.21, 95% CI 1.19-1.22) occurred in April-December 2020 compared with the April-December months in the pooled period 2016-2019. The SMR was more pronounced for men than for women (SMR 1.26, 95% CI 1.24-1.29 vs SMR 1.12, 95% CI 1.10-1.14), and it was statistically significant for both genders (*P*<.05). Using overdispersed Poisson generalized linear models, the number of expected deaths in April-December 2020 was 12,845 (7957 for women and 4888 for men). The total number of excess deaths during this period was estimated at 4583 (95% CI 4451-4716), with higher excess deaths in men (3112, 95% CI 3003-3221) than in women (1503, 95% CI 1427-1579). Almost 83.66% of excess deaths were attributed to COVID-19 in the Ministry of Health database. The vast majority of excess deaths occurred in people aged 60 years or older.

**Conclusions:**

The reported COVID-19 death counts underestimated mortality attributable to COVID-19. Excess deaths could reflect the increased deaths secondary to the pandemic and its containment measures. The majority of excess deaths occurred among old age groups. It is, therefore, important to maintain essential services for the elderly during pandemics.

## Introduction

The impact of COVID-19 and its response measures on health, economy, and society has been substantial. By the end of 2020, more than 380 million COVID-19 cases were confirmed worldwide, and more than 1.9 million deaths were attributed to COVID-19 [1]. In Jordan, the death toll has reached 3834, with more than 294,000 people diagnosed with COVID-19 at the end of 2020 [1]. The number of officially reported COVID-19 deaths in Jordan [2] does not reflect the true burden of the pandemic because some people with COVID-19 died without being diagnosed and because of the indirect impact of COVID-19 and its response measures.

All-cause mortality and estimates of excess deaths are commonly used in different countries to estimate the burden of COVID-19 and assess its direct and indirect effects [3,4]. However, the calculation of these measures is challenged by data gaps in some countries. Excess deaths are calculated by subtracting the number of expected deaths in a specific period from the number of observed deaths in the same period. In the context of COVID-19, the number of excess deaths refers to deaths that are directly or indirectly attributed to COVID-19. The indirect effects of the pandemic and its response measures result from denied or delayed diagnosis, management, and prevention of diseases; delayed care for acute emergencies; economic hardship; health care shortages; overburdened health care systems; disruption of essential health services; psychological distress; and domestic violence [5,6].

Previous studies used different methods to estimate excess deaths such as Farrington surveillance algorithms [7], the standardized mortality ratio (SMR) [8], the difference-in-differences econometric approach [9], generalized linear models such as Poisson loglinear and negative binomial with log link models [10], and the relevant excess mortality calculation method [11]. Many previous studies documented the excess mortality attributable to COVID-19. One study in the United States showed that COVID-19 deaths are likely to be twice as high as reported [12]. In Portugal, a study reported that excess deaths are 3-5 times higher than what could be explained by COVID-19 deaths [13]. Studies on the burden of COVID-19 in the Eastern Mediterranean region as well as in Jordan are scarce. This study aimed to analyze excess mortality during the COVID-19 pandemic in Jordan in April-December 2020.

## Methods

Official data on deaths in Jordan for 2020 and previous years (2016-2019) were obtained from the Department of Civil Status in March 2021. The data included information on age, sex, and date of death. Data on the number of officially registered COVID-19 deaths in Jordan were obtained from the Ministry of Health (MoH) [2]. The data on COVID-19 deaths were validated from different sources.

Mortality information was grouped by month to assess the temporal trends. Analysis was limited to April-December because the first COVID-19 death in Jordan occurred on March 28, 2020. Crude, gender-specific, and age-specific death rates were calculated. We contrasted mortality rates in 2020 with those in each year and the pooled period 2016-2020 using SMR by calculating the ratio between the observed number of deaths in Jordan in the year 2020 and the number of deaths that would be expected, based on the age- and sex-specific rates in 2016, 2017, 2018, 2019, and a pooled period 2016-2019. A ratio greater than 1.0 indicates excess deaths in the Jordan population in 2020. The 95% CI for SMR was calculated. The SMR was considered statistically significant if the value 1 was not included in the CI.

Expected deaths for 2020 were estimated by fitting the overdispersed Poisson generalized linear models to the monthly death counts for the period of 2016-2019. The model included month and year as variables to capture seasonality and adjust for annual trends. The month was entered in the model as a categorical variable. The model included age and gender as independent variables. Expected deaths were estimated for each gender-age stratum as a difference between observed and expected deaths. The total excess deaths was calculated by summing excess deaths across age categories for each gender. When the excess mortality in some age groups was less than 0 (indicating decreased deaths), the number of excess deaths was set as 0. Excess deaths are reported by gender, age group, and month.

## Results

A total of 22,429 deaths were registered in the Department of Civil Registration in April-December 2020. The total number of COVID-19 deaths registered by the MoH during the same period was 3834, accounting for 17.09% of total deaths. The gender-specific and age-specific death rates in April-December 2020 are shown in Table 1.

**Table 1:** The gender and age-specific death rates in Jordan (April-December 2020).

Age categories (years)	Women				Men		
	Population (n=5,084,000), n	Observed deaths (n=9051), n	Age-specific death rate (per 100,000 population)	Population (n=5,722,000), n		Observed deaths (n=13,378), n	Age-specific death rate (per 100,000 population)
<20	2,317,351	567	24	2,468,981		758	31
20-29	904,139	174	19	1,109,952		320	29
30-39	722,044	252	35	848,784		471	55
40-49	534,417	513	96	638,203		996	156
50-59	317,390	996	314	356,629		2034	570
60-69	168,889	1507	892	174,314		2698	1548
≥70	119,770	5042	4210	125,137		6101	4875

**Figure 1:**
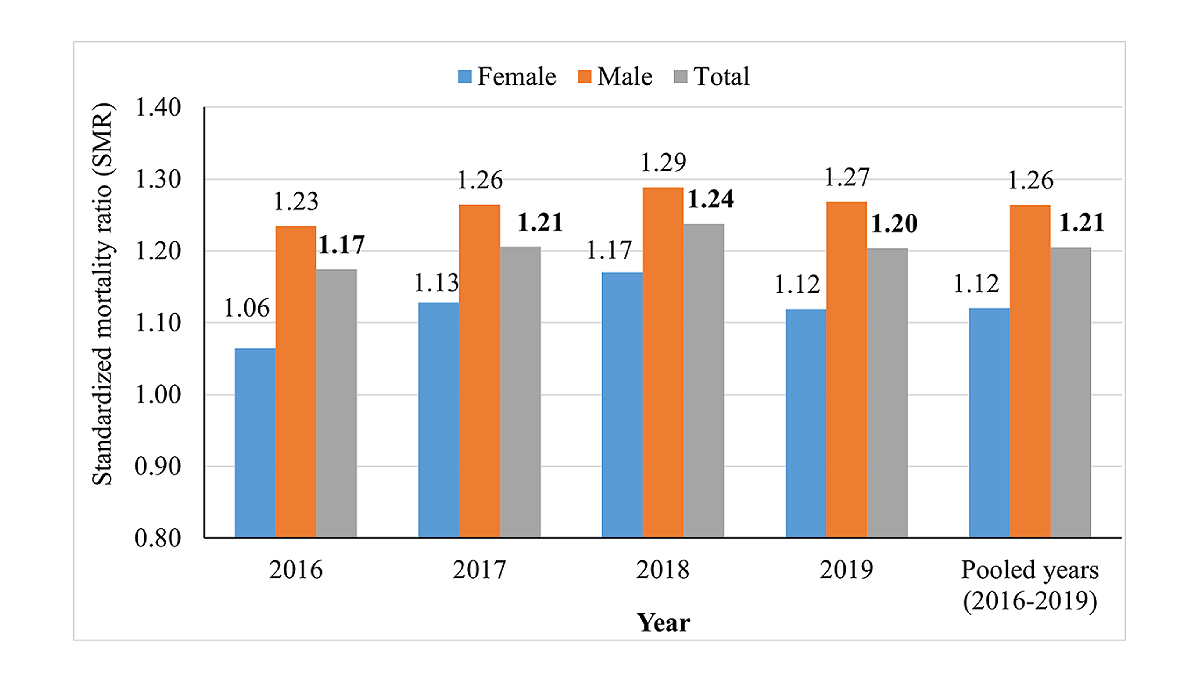
Comparison of the standardized mortality rate (SMR) between April-December 2020 and the same months in the years 2016, 2017, 2018, 2019, and the pooled years 2016-2019.

The number of observed deaths in April-December 2020 exceeded the average number of deaths in the period 2016-2019 by 28.0% (4902 deaths). To adjust for changes in age distribution over time and population growth, the SMR was calculated to compare the mortality in April-December 2020 with that in the same months of the years 2016, 2017, 2018, and 2019 and in the pooled years 2016-2019. Overall, a 21% increase in standardized mortality (SMR 1.21, 95% CI 1.19-1.22) occurred in April-December 2020 compared to average mortality in the period 2016-2019. The SMR was more pronounced for men than for women (SMR 1.26, 95% CI 1.24-1.29 vs SMR 1.12, 95% CI 1.10-1.14), and it was statistically significant for both genders (*P*<.05). Figure 1 shows the comparison of the SMR between April-December 2020 and April-December of the years 2016, 2017, 2018, and 2019 and pooled years 2016-2019.

**Figure 2:**
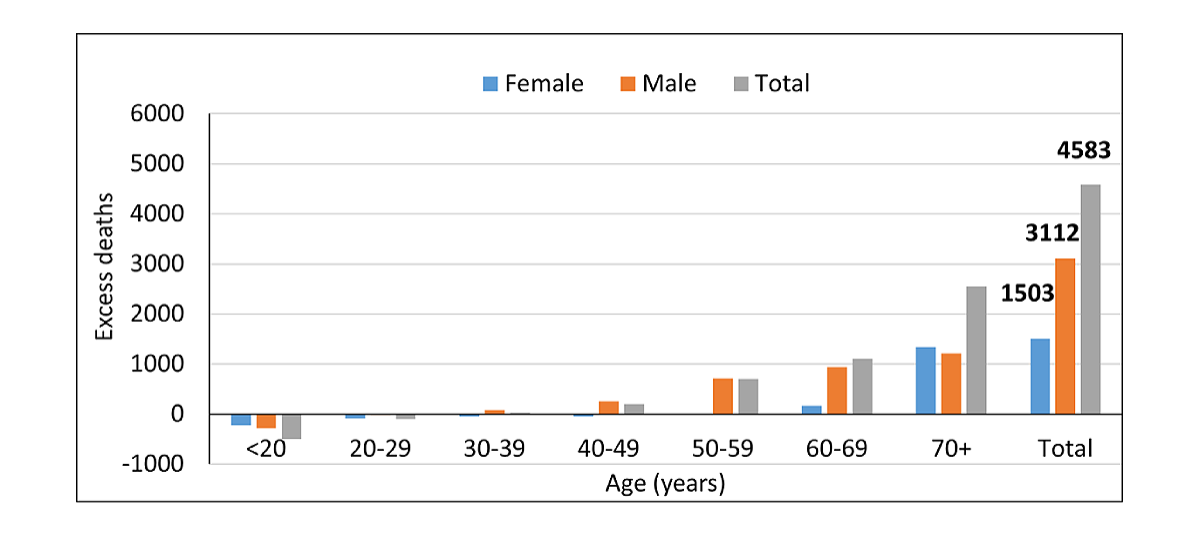
The number of excess deaths for men and women according to age group.

Using overdispersed Poisson generalized linear models to consider seasonal and temporal trends, the number of expected deaths in April-December 2020 was 12,845 (7957 in women and 4888 in men). The total number of excess deaths during this period was estimated at 4583 (95% CI 4451-4716), with higher excess deaths in men (3112, 95% CI 3003-3221) than in women (1503, 95% CI 1427-1579). Almost 83.66% (3834/4583) of excess deaths were attributed to COVID-19 in the MoH database. Figure 2 shows the number of excess deaths for men and women according to age group. The vast majority of excess deaths occurred in people aged 60 years or older (total: 3650/4583, 79.64%; women: 1503/1503, 100%; men: 2147/3112, 69.00%). It is worth mentioning that the numbers of deaths that occurred in women aged <60 years and in men aged <30 years in 2020 were less than expected.

**Figure 3:**
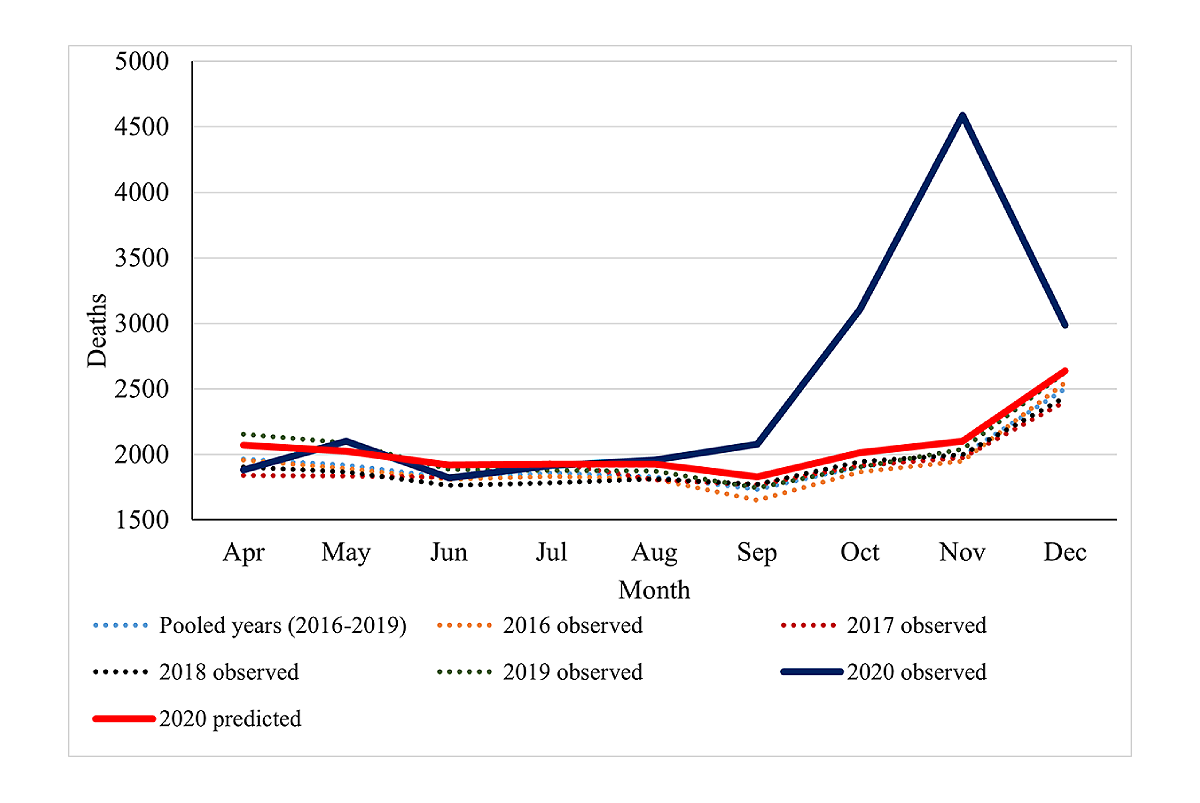
The number of observed deaths in 2016-2020 and predicted deaths in 2020 according to the month of the year.

Figure 3 shows the number of observed deaths in 2016-2020 and predicted deaths in 2020 according to the month of the year. The observed deaths mainly exceeded the expected deaths during the period September-December 2020 (Figure 3).

## Discussion

### Principal Findings

In this study, we compared the mortality experience in 2020 with that in previous years using indirect standardization of mortality rates and Poisson generalized linear models. We used standardized death ratios of mortality rates to adjust for the effects of differences in population age distributions over years. Age-specific mortality rates were also reported because only reporting standardized rates may mask the age differences in mortality. Poisson models were used to take into account the underlying annual and seasonal trends.

This study showed a 21% increase in standardized mortality in April-December 2020 compared with the same months in the pooled period of 2016-2019. Previous studies had documented increased mortality in some countries. One study in the United States [8] reported a 15.9% increase in the SMR in 2020 compared with 2019. In Switzerland, a study [14] showed that the SMR was 8.8% higher in 2020 than in 2019, returning to the level observed 5-6 years before around the year 2015. Todd et al [10] showed a 32% increase in deaths, which was above expectations, in Philadelphia during the period from March 22, 2020 to January 2, 2021. The differences in the estimates among different studies including ours might be explained by the differences in the onset of the epidemic in each country, the time at which each country started restrictive measures, and compliance rates.

In our study, higher excess deaths occurred among men and in people aged 60 years or older. The study in the United States [8] reported a sharp increase in mortality rates with increasing age, as well as rates that were higher in men than in women. The Swiss study [12] showed that the increase in deaths was greater for men than for women and was statistically significant only for men over 70 years of age and for women over 75 years of age. Todd et al [10] showed that excess mortality was disproportionately high among older adults. These findings highlight the need for separate analysis for gender- and age-specific strata to determine the groups at risk to be prioritized for interventions. Men were more affected than women, and older people were more affected than younger people.

In Jordan, deaths attributed to COVID-19 accounted for 83.66% of all excess deaths in 2020. In the United States, they accounted for about 75% of all excess deaths in 2020 [15,16]. Todd et al [10] showed that 77% of excess deaths were attributed to COVID-19 on death certiﬁcates.

Another finding in this study is that there was a greater reduction in the number of deaths in women aged <60 years and in men aged <30 years in 2020 than what would be expected. This finding might be explained by the reduction in deaths from other common causes in Jordan such as those caused by traffic accidents, due to lockdown and movement restrictions.

It is worth mentioning that the methodological approach we used can be used in other countries that have a strong death registration system. One should consider the limitation of excess death statistics when interpreting the study findings. This measure cannot be used to compare the burden of disease across countries because it depends on the size of the population [17]. Another point to consider is that mortality data might take some time before they become complete.

### Conclusion

The reported COVID-19 death counts in Jordan underestimated mortality attributable to COVID-19. The majority of excess deaths occurred among old age groups. Excess deaths could reflect increased deaths secondary to the pandemic and its containment measures. It is, therefore, important to maintain essential services for the elderly during pandemics.

## Abbreviations

MoH: Ministry of Health
SMR: standardized mortality ratio
